# Severe Hypercapnia in Critically Ill Adult Cystic Fibrosis Patients

**DOI:** 10.4021/jocmr612w

**Published:** 2011-09-26

**Authors:** Hassan S. Sheikh, Noel Dexter Tiangco, Christopher Harrell, Robert L. Vender

**Affiliations:** a Penn State Milton S. Hershey Medical Center, 500 University Drive, Hershey, PA 17033, USA

## Abstract

**Background:**

Cystic fibrosis (CF) is a monogenetic autosomal recessive multi-organ disease affecting approximately 50,000 patients worldwide. Overall median survival is continually increasing but pulmonary disease remains the most common cause of death. Guidelines have been published in relation to the outpatient maintenance of lung health for CF patients and treatment of acute lung exacerbations but little information exists about the management of the critically ill CF patient. Invasive mechanical ventilation in CF patients with acute respiratory failure is associated with poor outcome and high mortality.

**Methods:**

Retrospective analysis of adult patients with CF who required endotracheal intubation and invasive mechanical ventilation in the Medical Intensive Care Unit (MICU).

**Results:**

Between the years 2003 - 2009, 14 adult patients with CF required endotracheal intubation and invasive mechanical ventilation in the Medical Intensive Care Unit (MICU) of the Penn State Milton S. Hershey Medical Center, Hershey, Pennsylvania, USA. Eleven patients died in the MICU because of progressive respiratory failure and inability to liberate from mechanical ventilation. Seven individuals consistently manifested arterial partial pressures of carbon dioxide (PaCO_2_) greater than 20.00 kPa despite high levels of conventional modes of mechanical ventilation.

**Conclusion:**

Intubated CF patients with respiratory failure have a high mortality rate. Based on our experience, multiple factors contribute to severe hypercapnia and the effectiveness of conventional modes of mechanical ventilation in many of these patients is limited.

**Keywords:**

Cystic fibrosis; Mechanical ventilation; Critical care; Hypercapnia; Respiratory failure

## Introduction

Cystic fibrosis (CF) is a monogenetic autosomal recessive multi-organ disease affecting approximately 50,000 patients worldwide. Despite the increasing longevity and improving median age of survival of CF patients, for the vast majority, CF remains a fatal disease. As the CF population becomes older and especially with advancements in lung transplantation, more patients are being admitted and cared for in critical care settings. Unfortunately, particularly for those requiring endotracheal intubation and invasive mechanical ventilation for respiratory failure, the outcome continues to be poor [1-3].

Little information exists in relation to specific aspects of the intensive care management of these complex patients, especially with regard to invasive mechanical ventilation [4]. This is in contrast to pulmonary entities such as asthma, chronic obstructive pulmonary disease (COPD), and acute respiratory distress syndrome (ARDS) for which ventilator strategies have been recommended, based on the current understanding of their underlying pathophysiologic processes, with the aim of providing adequate gas exchange while minimizing adverse effects [5,6]. In this paper, we present our experience with CF patients requiring invasive mechanical ventilation, hoping to expand the understanding of these critically ill patients.

## Materials and Methods

In a retrospective chart review, 7 adult patients with CF who were endotracheally intubated and mechanically ventilated in the Medical Intensive Care Unit (MICU) of the Penn State Milton S. Hershey Medical Center within the last 7 years were selected. Demographic information, arterial blood gas (ABG) data, and respiratory-related parameters were collected. ABG measurements were obtained from samples of heparinized arterial blood and partial pressures of carbon dioxide (PaCO_2_) and oxygen (PaO_2_) measured using Automated Instruments with a reported linear range of accuracy for PaCO_2_ as high as 33.33 kPa. Dead space fraction was estimated for each patient using measured PaCO_2_ and minute ventilation (V_E_) with the assumption that CO_2_ production (VCO_2_) for each was 250 mL/min [7,8]. All laboratory determinations were made during periods of direct observation at times of worsening clinical status in hospitalized patients in a monitored environment. None of the evaluations were obtained at a time of unexpectedly finding any patient in a ”code“ situation or clinically unanticipated deterioration. This study was performed in full compliance with regulations and requirements for human investigation in relation to decedents’ protected health information through the Human Subjects Protection Office of the Penn State Milton S. Hershey Medical Center.

## Results

From 2003 to 2009, 14 adults (age 18 years or older) with CF requiring endotracheal intubation and invasive mechanical ventilation were admitted to the MICU at the Penn State Milton S. Hershey Medical Center. All patients received some mode of standard conventional ventilation: intermittent mandatory ventilation-pressure control (IMV-PC) including Airway Pressure Release Ventilation (APRV), continuous mandatory ventilation-pressure control (CMV-PC), intermittent mandatory ventilation-dual control (IMV-DC), and continuous mandatory ventilation-dual control (CMV-DC) [9]. The dual control modes included both pressure regulated volume control (PRVC) and autoflow. These 14 patients accounted for 364 MICU days and 309 ventilator days. Eleven patients died during their MICU stay because of progressive respiratory failure and inability to liberate from mechanical ventilation, 1 patient died from septic shock secondary to acute pyelonephritis, 1 patient was discharged to home hospice care and subsequently died, while another survived and was discharged home in improved condition.

The 11 patients who died from respiratory causes and the lone survivor demonstrated marked elevations in PaCO_2_. Furthermore, 7 of those patients consistently manifested PaCO_2_ measurements above 20.00 kPa (range 22.66-33.06 kPa). The 7 patients had been admitted to the ICU because of hypercapnic respiratory failure secondary to an acute infectious pulmonary exacerbation. All 7 died after medical support was withdrawn in compliance with requests of family. Patient demographics and physiological information of these patients are reported in Table 1.

**Table 1:** Demographics and Physiological Information

Number	Age	Gender	Weight	BMI	FEV1			Respiratory Pathogens	Total Days Vent	V_E_	Calculated Vd/Vt	Critical ABG						
	(years)		(kg)	(kg/m^2^)	(L)		(% predicted)			(L/min)		pCO_2_		pH		pO_2_		HCO_3_
1	18	F	38.8	13.8	1.83		0.53	BC, PA	4	14.4	0.92	179		6.87		55		32
2	24	F	45.2	16.2	0.69		0.24	PA, MRSA	46	NA	NA	224		6.94		106		47
2										NA	NA	178		6.95		104		38
2										6.2	0.83	206		7.07		50		57
2										9	0.86	170		7.09		86		51
3	24	F	39.2	16	0.46		0.14	PA, MRSA	28	9.9	0.9	209		6.87		72		37
4	27	F	37.9	15.2	1.15		0.39	BC	39	3.7	0.73	218		6.95		84		47
5	28	F	47	18.2	1.16		0.4	BC, MRSA	12	10.3	0.88	172		7.08		80		50
6	33	F	42.4	16.8	0.82		0.31	PA, MRSA	12	4.6	0.81	241		6.86		150		42
6										4.5	0.81	248		6.86		114		43
6										4	0.77	234		6.83		96		38
7	35	M	53.4	15.9	0.6		0.13	BC	5	10.2	0.9	230		6.75		134		30
7										7.2	0.86	211		6.77		121		30
7										8.5	0.78	203		6.82		151		32

BMI: body mass index; FEV1: forced expiratory volume in one second; BC: Burkholderia cepacia complex; PA: Pseudomonas aeruginosa; MRSA: Methicillin-resistant Staphylococcus aureus; VE: minute ventilation; Vd/Vt: ratio of dead space ventilation to tidal volume; NA: not available.

## Discussion

While prior studies have shown that non-invasive ventilation has decreased the mortality rate of critically ill CF patients, survival following institution of invasive ventilation remains poor [1-3]. Our findings are in agreement with the reported high mortality associated with invasive ventilation. Other predictors of poor outcome include rapid decline in FEV1 leading up to the time of ICU admission, prior *Burkholderia cepacia* complex lung infection, and the severity of the acute exacerbation [10]. Consistent with previous publications, female gender, malnutrition, and *B. cepacia* syndrome were also significant factors associated with a fatal outcome [11,12]. Potentially reversible complications of CF-lung disease such as spontaneous pneumothorax or massive hemoptysis do not invariably portend a predictable fatal outcome but were not identified in the patients in this study [11].

The magnitude of elevations in PaCO_2_ for these 7 reported patients far exceeds measurements previously reported in either patients with CF or COPD even during acute exacerbations [13]. In fact there is only a single notation of three patients with PaCO_2_ measurements above 14.40 kPa in a review that included 65 admissions to the ICU for respiratory failure, 32 of them requiring invasive mechanical ventilation [11]. In addition, a second study of only 8 adult patients with CF who required invasive ventilation reported a mean PaCO_2_ of 13.60 ± 8.00 kPa [1].

The 7 patients in this paper demonstrated severe hypercapnia. These findings are likely contributed to by several factors: First, dead space fraction was markedly increased for every patient. Calculated ratios of dead space ventilation to tidal volume (Vd/Vt) consistently exceeded 70%, a level of abnormality predictive of failure to maintain spontaneous ventilation and adequate gas exchange without mechanical ventilatory support [14,15]. Second, hyper-catabolism related to sustained endobronchial lung suppuration and high levels of respiratory muscle recruitment increases CO_2_ production [16,17]. Third, the strikingly high levels of hypercapnia indicate the difficulty we had providing ample ventilation to these patients. High airway resistance secondary to secretions, airway inflammation, and obliterated bronchi leads to elevated peak pressures, a tendency for air trapping, and ultimately, inadequate alveolar ventilation [16].

The clinical management of CF is unique, complicated and multi-faceted. Once entering adulthood, CF patients also maintain autonomy in deciding the extent and limits of their health care and treatments. The purpose of our review was not to debate the merits or ethics of acute invasive mechanical ventilation in CF patients with severe chronic end organ disease but rather to highlight specific clinical observations of critically ill CF patients, all of whom died in the MICU [18,19].

Given the consistently reported high mortality of critically ill adult CF patients requiring invasive mechanical ventilation, it is of paramount importance to institute timely and optimal management and support of organ systems failure while acknowledging the absence of evidence-based practice guidelines. This is especially important when the initiation of invasive mechanical ventilation is used as a bridge to lung transplantation.

Until studies are performed that better define the complex gas exchange in acute respiratory failure in CF and an approach to invasive ventilation for CF patients requiring this therapy is validated, awareness of these observations should assist current clinical practice guidelines.
